# A stepwise data interpretation process for renal amyloidosis typing by LMD-MS

**DOI:** 10.1186/s12882-022-02785-9

**Published:** 2022-04-13

**Authors:** Ming Ke, Xin Li, Lin Wang, Shuling Yue, Beibei Zhao

**Affiliations:** Guangzhou KingMed Center for Clinical Laboratory Co.,Ltd, Guangzhou, 510005 China

**Keywords:** Amyloidosis, Laser microdissection, Mass spectrometry, Proteomics

## Abstract

**Backgrounds:**

Systemic amyloidosis is classified according to the deposited amyloid fibril protein (AFP), which determines its best therapeutic scheme. The most common type of AFP found are immunoglobulin light chains. The laser microdissection combined with mass spectrometry (LMD-MS) technique is a promising approach for precise typing of amyloidosis, however, the major difficulty in interpreting the MS data is how to accurately identify the precipitated AFP from background.

**Objectives:**

The objective of the present study is to establish a complete data interpretation procedure for LMD-MS based amyloidosis typing.

**Methods:**

Formalin-fixed paraffin-embedded specimens from patients with renal amyloidosis and non-amyloid nephropathies (including diabetic nephropathy, fibrillary glomerulonephritis, IgA nephropathy, lupus nephritis, membranous nephropathy, and normal tissue adjacent to tumors) were analyzed by LMD-MS. Forty-two specimens were used to train the data interpretation procedure, which was validated by another 50 validation specimens. Area under receiver operating curve (AUROC) analysis of amyloid accompanying proteins (AAPs, including apolipoprotein A-IV, apolipoprotein E and serum amyloid P-component) for discriminating amyloidosis from non-amyloid nephropathies was performed.

**Results:**

A stepwise data interpretation procedure that includes or excludes the types of amyloidosis group by group was established. The involvement of AFPs other than immunoglobulin was determined by P-score, as well as immunoglobulin light chain by variable of λ-κ, and immunoglobulin heavy chain by H-score. This achieved a total of 88% accuracy in 50 validation specimens. The AAPs showed significantly different expression levels between amyloidosis specimens and non-amyloid nephropathies. Each of the single AAP had a AUROC value more than 0.9 for diagnosis of amyloidosis from non-amyloid control, and the averaged level of the three AAPs showed the highest AUROC (0.966), which might be an alternative indicator for amyloidosis diagnosis.

**Conclusions:**

The proteomic data interpretation procedure for LMD-MS based amyloidosis typing was established successfully that has a high practicability in clinical application.

**Supplementary Information:**

The online version contains supplementary material available at 10.1186/s12882-022-02785-9.

## Background

Systemic amyloidosis is a group of heterogeneous diseases caused by protein structural abnormality. The pathological character of amyloidosis is the formation of extracellular deposition of beta-sheet fibril through aggregation of insoluble proteins or peptides. The cytotoxicity of the deposited proteins cause destruction of tissue and cellular structure, which then induce functional injury of organs, such as kidney [1–3]. Systemic amyloidosis is classified according to the type of deposited protein, these mainly include immunoglobulin light/heavy chain, and AFPs other than immunoglobulin [3–8]. The major affected organs, prognosis and effective treatment strategies of each amyloidosis type are different [9–12], therefore, precise typing is of paramount importance for providing patients with the most appropriate care.

For diagnosis of amyloidosis, histopathologic examination methods are mostly used in routine practice, such as histochemical stain by Congo red (CR) [13]. Further typing methods are usually based on immunohistochemistry and immunofluorescence, which rely on antibody recognition that might be interfered by background and epitope loss, and could not identify new AFPs [14]. In 2008, Mayo Clinic proposed the LMD-MS technique for amyloidosis typing [15], and it gradually became the gold standard in recent years [3]. MS-based assay possesses cost advantage for it is a test once for all proteins, whereas antibody-based immunoassay is a test once for only one protein [16, 17].

However, there is still a gap between the LMD-MS technology itself and its transition to clinical application, that is largely due to the uncertainty of how to interpret the MS data. As proposed by Mayo Clinic, the type of amyloidosis is called by considering the most abundant amyloid protein that has the maximal MS/MS spectral count (SC) (Mayo’s rule) [18–20]. Nevertheless, in routine practical detection, multiple AFPs are often identified at the same time. The pathogenic protein may not have an absolute preponderance of SC when compared with other AFPs in background for: 1) the deposition degree of the pathogenic amyloid protein is not much significantly superior to others, or 2) blood contamination happens, or 3) little amount of material sampled from microdissection. This makes it challenging to make a precise typing by simply extracting the protein with the highest SC. For instance, IgG is often identified with very high SC in immunoglobulin light chain amyloidosis (AL) [21]. In Leukocyte chemotactic factor-2 amyloidosis (ALECT2), empirical data showed that the SC of LECT2 is generally low, and is almost impossible to be the highest [22]. Furthermore, this relative quantification of protein SCs within a specimen cannot be extended to comparing different samples. Thus, it may need a global normalization method for establishing a relatively unified quantitative analysis system.

The prevalence of different amyloidosis types varies greatly in different regions, however, AL is always the most frequent one (59%-68%); serum amyloid A amyloidosis (AA, 4–12%), ALECT2 (~ 3%) and transthyretin amyloidosis (ATTR, 3%-33%) could be considered as less frequent types; whereas the other types are rare [1, 23, 24]. In AL, κ-type (ALκ) and λ-type (ALλ) interfere with each other, mainly because of the high background of κ chain. In terms of typing fibrinogen A α chain amyloidosis (AFib), the SC of FIBA should be greater than the sum of fibrinogen B β chain (FIBB) and fibrinogen G γ chain (FIBG) in addition to having the highest SC among all AFPs [25]. Besides, special considerations are required for making definite diagnosis of immunoglobulin heavy chain amyloidosis (AH) and its involvement with AL, that is immunoglobulin heavy-light chain amyloidosis (AHL) [26]. Thus, it is reasonable to propose a stepwise process to include or exclude specific amyloidosis types. In this study, we aimed to establish an LMD-MS based amyloidosis typing procedure, especially for the rules of MS data interpretation.

Another important aspect of LMD-MS based amyloidosis typing is quantifying accompanying proteins (AAPs) that are co-deposited with amyloid fibrils. As far as we know, three proteins named apolipoprotein E (APOE), apolipoprotein A-IV (APOA4), and serum amyloid P component (SAP) are usually accompanied with amyloid deposition in amyloidosis. Although the LMD-MS analysis is usually executed based on histopathologic diagnosis, that is usually a positive staining of CR, the three AAPs are often regarded as the evidence of amyloid deposition. Even, the criterion of detection of at least two of the three AAPs has been surrogate for CR staining [27]. However, whether these three AAPs’ quantitative information is valuable for improving amyloidosis diagnosis needs further investigation.

## Methods

### Clinical specimens

A total of 92 formalin fixed paraffin-embedded (FFPE) renal puncture specimens of systemic amyloidosis that were diagnosed in the Department of Pathology, Guangzhou KingMed Diagnostics were collected, of which 42 retrospective specimens received from January 2018 to December 2019 were used for method training, and 50 from January 2020 to June 2021 were collected for method validation. These specimens were required to meet the following criteria: CR staining was positive and apple-green birefringence was observed under polarized light; unbranched and randomly arranged fibrous at 8-12 nm under electron microscopy; CR positive fibrillary glomerulonephritis was excluded by the marker DNAJB9 [28]. The amyloidosis types of the cases were characterized by immunofluorescence, immunohistochemistry, immuno-electron microscopy, serum and urine test of immunofixation electrophoresis or serum free light chain. The results were reviewed by three senior licensed pathologists. We also collected 55 other specimens that were non-amyloid nephropathies as control. This study has been approved by the Ethics Committee of Guangzhou KingMed Diagnostics and meets the ethical requirements.

### Specimen preparation and laser microdissection

FFPE tissue specimen was cut into 7 μm sections, which were then transferred onto a specialized POL-membrane for microdissection on a steel frame slide. Sections were then air dried, melted, and deparaffinized. After that, CR staining was conducted to identify positive areas of amyloid deposition. The selected CR positive micro area were dissected using the Leica LMD6 system. A 0.5 mL centrifuge tube prefilled with 40 μL lysate buffer (10 mM Tris, 1 mM EDTA, 0.5% NaDOC) on the cap was used to collect the micro pieces. A total of at least 200 000 μm^2^ dissection area of each specimen was required.

### Peptide sample preparation

The collected micro piece was centrifuged to the bottom of the tube, followed by ultrasonication for 15 min, incubation at 98 ℃ for 1 h to de-crosslink proteins, and ultrasonication again for 15 min. Protein was then digested by 0.5 ug trypsin (Promega) at 37℃ for 4 h or overnight. The generated peptide was reduced by 5 mM dithioethylitol at 37℃ for 30 min, and alkylated by 15 mM iodoacetamide at room temperature in dark for 45 min. Trifluoroacetic acid was added to terminate the reaction, followed by centrifugation at 20 000 g, the supernatant was collected. The Ziptip C18 column (Millipore) was used for desalination of the peptide solution. The eluted peptide mixture was then dried by a vacuum-frozen concentrator and redissolved with 0.1% trifluoroacetic acid in water before MS analysis.

### LC–MS/MS analysis and data retrieval

The peptide mixture was subjected to the Ultimate 3000 RSLC nanoLC, separated by online reversed-phase chromatography, and then injected into the Q Exactive mass spectrometer (Thermo Scientific) via Nano-ESI source. MS data was firstly converted to mgf file by ProteoWizard and then retrieved by Mascot software (Matrix Science), using the protein database of Home sapiens from Swissport. The search parameters were set as follows: Enzyme: Trypsin/P; Allow up to: 2 missed cleavages; Fixed modifications: Carbamidomethyl (C); Variable modifications: Oxidation (M); MS tol.: 10 PPM, MS/MS tol.: 0.02 Da. The PSM (peptide-spectrum-match) hit was re-scored by Percolator, and the retrieval results were filtered using the Proteome Discoverer software (Thermo) under the following conditions: FDR < 1%, Number of peptides matched ≥ 2.

### Protein quantification and statistical analysis

The relative abundance of a target protein was evaluated by its normalized SC, which was calculated through the following formula: ${\rm N}C_{i} = C_{i} /\sum\nolimits_{k = 1}^{n} {C_{k} } \times 1000$. Where NCi is the normalized SC of protein i, Ci is the absolute SC in MS raw data, and n is the number of all identified proteins [28]. The abundance of amyloid proteins was calculated accordingly. To calculate the superiority of the pathogenic protein over others, the superiority score (S-score) of each of the AFPs was defined as: $S - score_{i} = NC_{i} /Max(NC_{ - i} )$. Where Max(NC_-i_) was the maximum normalized SC of other AFPs than protein i. The λ-κ value for judging immunoglobulin light chain involvement was calculated by: $\lambda - \kappa = NC_{Ig\lambda } - NC_{Ig\kappa }$. And the superiority of immunoglobulin heavy chain over light chain (H-score) was defined as: $H - score = NC_{j} /Max(NC_{Ig\kappa } ,NC_{Ig\lambda } )$. Where protein j was limited to IgG, IgA or IgM. Wilcoxon rank test was used to analyze the significance of difference between paired SC of Igκ and Igλ within a group, and Mann–Whitney U test was used to analyze the significance of difference between groups.

## Results

### Demographics of the study cohort

To establish the amyloidosis typing procedure, FFPE tissue specimens from 42 renal amyloidosis patients were collected, including 9 ALκ, 12 ALλ, 4 ALECT2, 3 of each of AHL (IgGλ) and AHL (IgAλ), 2 cases of each of AH (IgG), AA and gelsolin amyloidosis (AGel), and 1 case of each of AHL (IgGκ), AFib, ATTR, apolipoprotein A1 amyloidosis (AApoAI) and lysozyme amyloidosis (ALys)). A total of 50 validation cases were used for testing the performance of the procedure. Among them, 13 and 17 subjects were ALκ and ALλ, as well as 5 cases of AHL (IgGλ), 3 cases of each of AH (IgG), AGel, ALECT2 and AHL (IgAλ), 2 cases of AA and 1 case of ATTR (Table 1). Additionally, the study included a set of control specimens that are non-amyloid nephropathy (diabetic nephropathy (DN, *n* = 13), fibrillary glomerulonephritis (FGN, *n* = 8), IgA nephropathy (IgAN, *n* = 9), lupus nephritis (LN, *n* = 5), membranous nephropathy (MN, *n* = 15) and normal tissue adjacent to tumors (NATs, *n* = 5). The clinical information for the subjects is listed in Table 2.

**Table 1:** Clinically confirmed types of training and validation dataset

Amyloidosis type	Training set	Validation set
ALκ	9	13
ALλ	12	17
AHL (IgGκ)	1	0
AHL (IgGλ)	3	5
AHL (IgAλ)	3	3
AH (IgG)	2	3
AA	2	2
AGel	2	3
ALECT2	4	3
ATTR	1	1
AFib	1	0
AApoAI	1	0
ALys	1	0
Total	42	50

**Table 2:** Clinic Characteristics of the Subjects

	Training set	Validation set	Non-amyloid nephropathy					
			DN	FGN	IgAN	LN	MN	NAT
Age (years)	60.0 (52.0–64.0)	61.0 (51.8–67.3)	56.0 (51.0–60.0)	43.0 (39.0–46.8)	41.0 (31.0–42.0)	34.0 (29.0–38.0)	51.0 (45.0–56.0)	-
Gender (male/female)	22/20	31/19	6/7	2/6	3/6	0/5	12/3	5^a^
Upro (mg/day)	3482 (1731–7490)	3716 (2755–5687)	3835 (2658–8013)	3480 (2740–5340)	1060 (815–1690)	3583 (2142–6214)	3984 (3720–4248)	-
Alb (g/L)	22.9 (17.8–28.3)	25.7 (20.7–29.3)	27.4 (25.1–32.2)	31.8 (28.9–33.4)	38.8 (38.7–39.0)	26.2 (24.0–29.7)	16.8 (15.8–17.7)	-
Scr (μmol/L)	84.0 (58.0–141.0)	85.0 (68.5–100.0)	108.0 (60.0–191.0)	64.0 (57.0–99.0)	82.0 (67.0–92.0)	64.0 (59.0–68.0)	93 (85.5–100.5)	-
BUN (mmol/L)	6.0 (5.4–9.0)	7.3 (4.5–9.2)	5.4 (4.4–7.7)	4.7 (3.3–6.2)	5.6 (5.2–7.0)	5.4 (3.6–5.5)	5.0 (3.4–5.8)	-

^a^Clinical information of NAT specimen was not collected

Data are presented as median with interquartile range

*Upro* urine protein, *Alb* albumin, *Scr* serum creatinine, *BUN* blood urea nitrogen

### Characteristics of spectral count distribution in training dataset

#### ALECT2

**Fig. 1:**
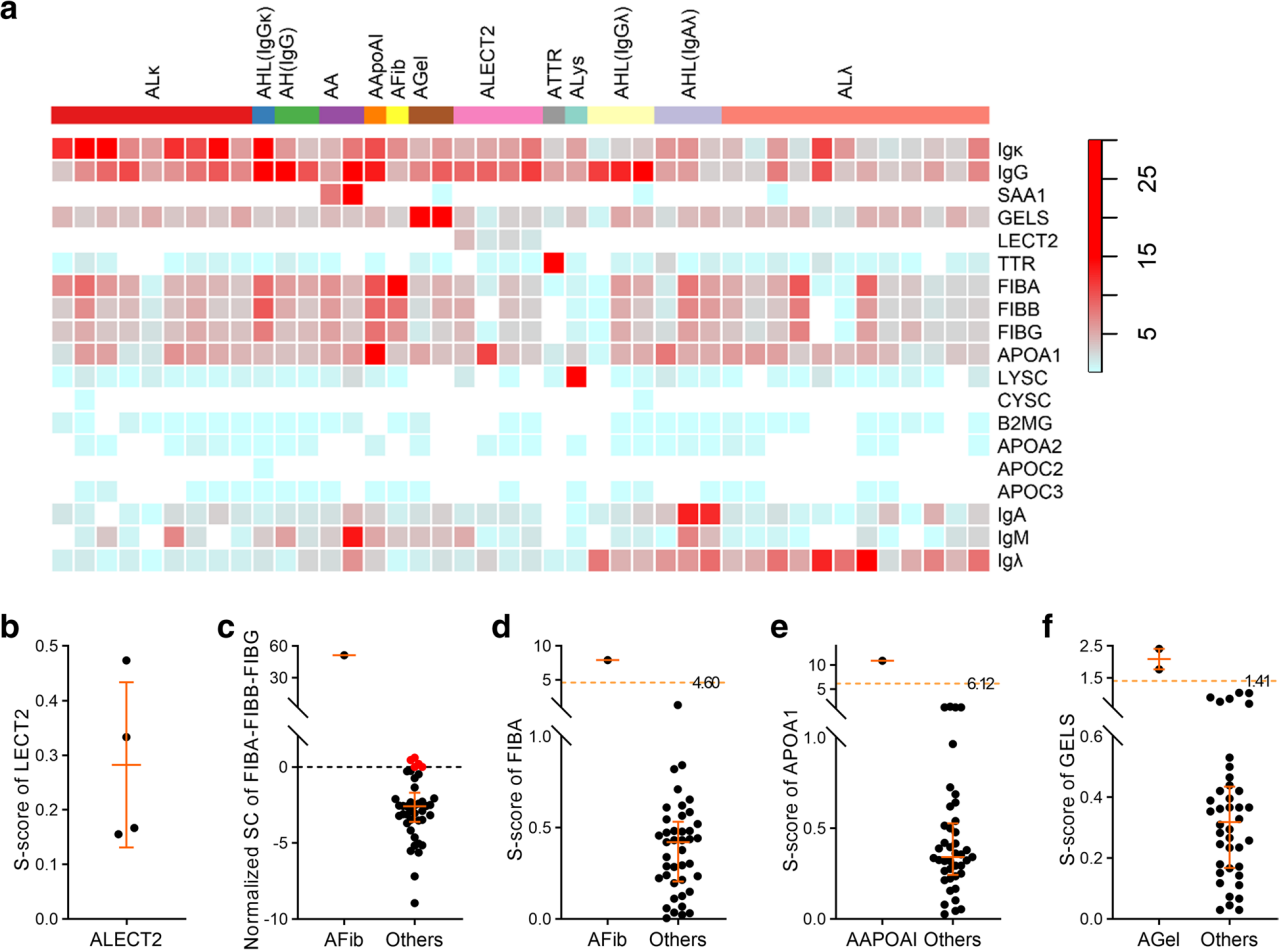
AFP’s quantitative profile and analysis of irregular ones. **a.** Normalized SCs of all the AFPs in each training specimen are presented as heatmap. Amyloidosis type of each case is labeled by column, and protein name is labeled by row. **b.** Distribution of LECT2’s S-score in ALECT2 cases. **c.** Scatter plot of normalized SC of FIBA minus the sum of FIBB and FIBG in AFib and types other than AFib. **d-f.** Comparison of S-scores of FIBA (**d**), APOA1 (**e**) and GELS (**f**) between these AFPs’ related and unrelated types. Median and interquartile range of the data is plotted

ALECT2 is quite special for it could be principally determined as long as LECT2 is identified at the deposition site [14]. Our result also showed that LECT2 only presented in ALECT2 cases with relatively lower abundance as compared to other amyloid proteins, and was almost absent from specimens other than ALECT2 (Fig. 1a). The S-score of LECT2 in ALECT2 was just a little bit beyond 0, but none of them was above 1 (Fig. 1b). Therefore, the identification or failure to identify LECT2 could be used as a criterion to diagnose or exclude ALECT2, but do not require having the highest SC.

#### AFib/AApoAI/AGel

The SCs of FIBA, apolipoprotein A-I (APOA1) and GELS were observed to be relatively high in the background across the training specimens (Fig. 1a and Supplementary Fig. 1a, c, e). This may interfere the identification of other types if the Mayo’s rule is utilized (Supplementary Fig. 1b, d, f). Consistent with previous report [25], the SC of FIBA was found to be significantly greater than the sum of FIBB and FIBG in AFib, while a few cases other than AFib have slightly higher SC of FIBA than the sum of FIBB and FIBG (Fig. 1c). The S-score of FIBA in AFib type was 7.92, but in cases other than AFib, it had a median of 0.42 (IQR, 0.22—0.52), and the cut-off value could be set to 4.60, which was the mean of the highest score in cases other than AFib (1.28) and the score in AFib (7.92) (Fig. 1d). Similarly, the SC of APOA1 was surprisingly high in AApoAI (Supplementary Fig. 1c). The median S-Score of APOA1 was 0.34 (IQR, 0.25—0.52) in cases other than AApoAI, whereas in AApoAI it was 10.96, the cut off could be 6.12 accordingly (Fig. 1e). Whereas for AGel type, the S-score of GELS in training set were 2.41 and 1.77, which were significantly higher than in cases other than AGel, 0.32 (IQR, 0.17—0.43), and the cut off could be 1.41 (Fig. 1f).

#### AA/ATTR/ALys

Serum amyloid A-1 (SAA1) was seldomly observed in cases other than AAtype, while transthyretin (TTR) and lysozyme C (LYSC) were identified frequently across the training specimens (Fig. 1a). Anyhow, these three amyloid proteins each showed evident SC superiority than others in their related amyloidosis type, and the opposite inferior position in their unrelated types. These results indicated that these three types could be determined via the Mayo’s rule (Supplementary Fig. 2a-c).

#### ACys/Aβ2M/AApoAII/AApoCII/AApoCIII

Although there was no specimen corresponding to the five rare types of ACys, Aβ2M, AApoAII, AApoCII and AApoCIII in the present study, we analyzed the characteristics of the corresponding AFPs’ SC distribution (CYSC/B2MG/APOA2/APOC2/APOC3). These proteins all showed very low abundance across the specimens, especially for CYSC and APOC2 were only identified twice and once respectively (Fig. 1a). Thus, it was assumed that these types may also follow the Mayo’s rule. However, we cannot exclude the possibility that they are similar to the evaluation method of ALECT2 in alternative.

### Immunoglobulin light chain-related amyloidosis (AL/AHL)

**Fig. 2:**
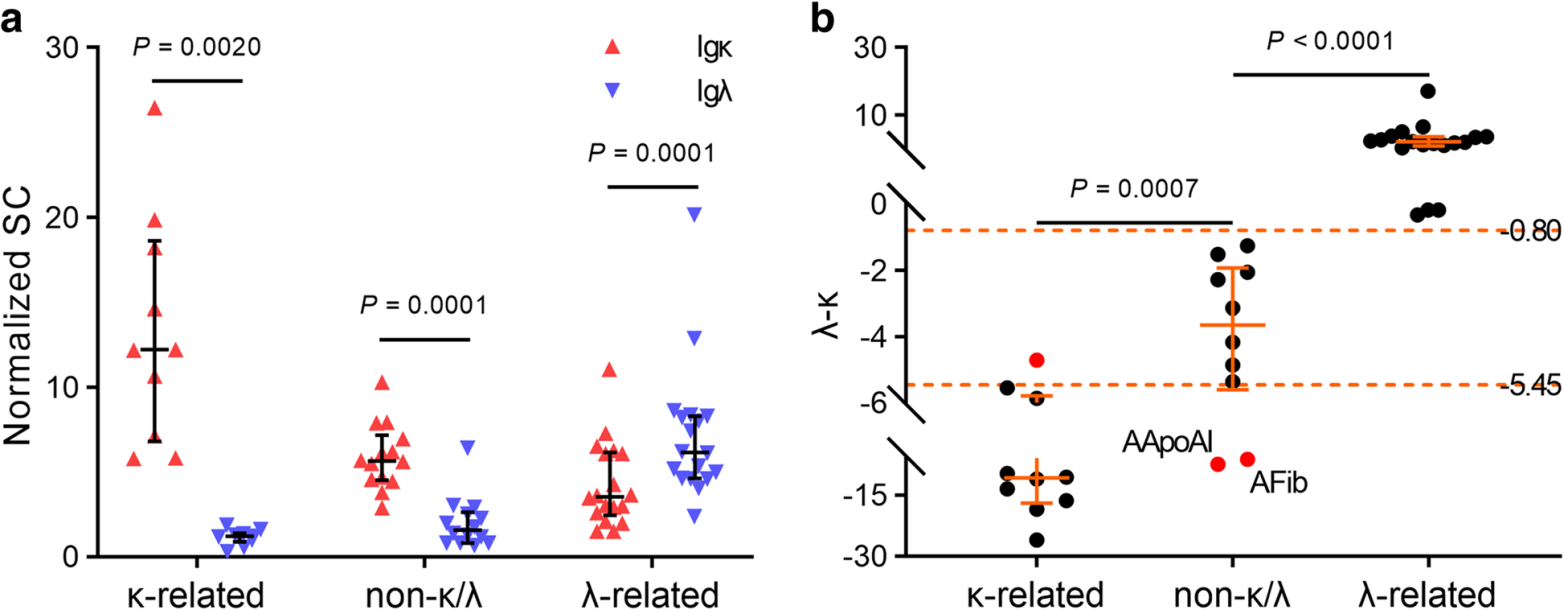
Analysis of the involvement of immunoglobulin light. **a.** Comparison of Igκ and Igλ’s normalized SCs within κ-related, λ-related or non-κ/λ groups, two-sided P values from paired Wilcoxon rank test are labeled. **b.** Comparison of λ-κ value among κ-related, λ-related and non-κ/λ groups, two-sided *P* values from Mann–Whitney U test are labeled. Median and interquartile range of the data is plotted

In Igκ-related amyloidosis (ALκ and AHL (IgGκ)), the SC of Igκ was almost always the highest except in one case that was exceeded by FIBA, and was always higher than Igλ. Obvious preponderance of Igκ SC over Igλ in neither Igκ- nor Igλ-related cases was also observed. In Igλ-related amyloidosis (ALλ and AHL (IgGλ/IgAλ)), the SC of Igλ has not such significant superiority as Igκ in Igκ-related amyloidosis, it was even sometimes less than Igκ, FIBA or APOA1 (Figs. 1a, and 2a). Thus, the specimens (ALECT2 cases were excluded) were then divided into three groups: κ-related, including ALκ and Igκ related AHLs; λ-related, including ALλ and Igλ related AHLs; and non-κ/λ related groups, for comparison of Igλ SC minus Igκ SC (λ-κ). As shown in Fig. 2b, there was significant difference of λ-κ between any two of the three groups. In κ-related group, the median value of λ-κ was -10.80 (IQR, -15.60—-6.81); in non-κ/λ group, it was -3.65 (IQR, -5.24—-2,12); In λ-related group, it was 2.02 (IQR, 0.97—3.48). There were two cases with the lowest λ-κ value that may interfere the judging of Igκ’s involvement, they were an AApoAI and an AFib respectively. After removing these two cases, the cut-off value could be set to -5.45 to that has the highest sensitivity and specificity in discriminating Igκ’s involvement. For judging Igλ’s involvement, the cut-off value could be -0.80.

### Immunoglobulin heavy chain-related amyloidosis (AH/AHL)

**Fig. 3:**
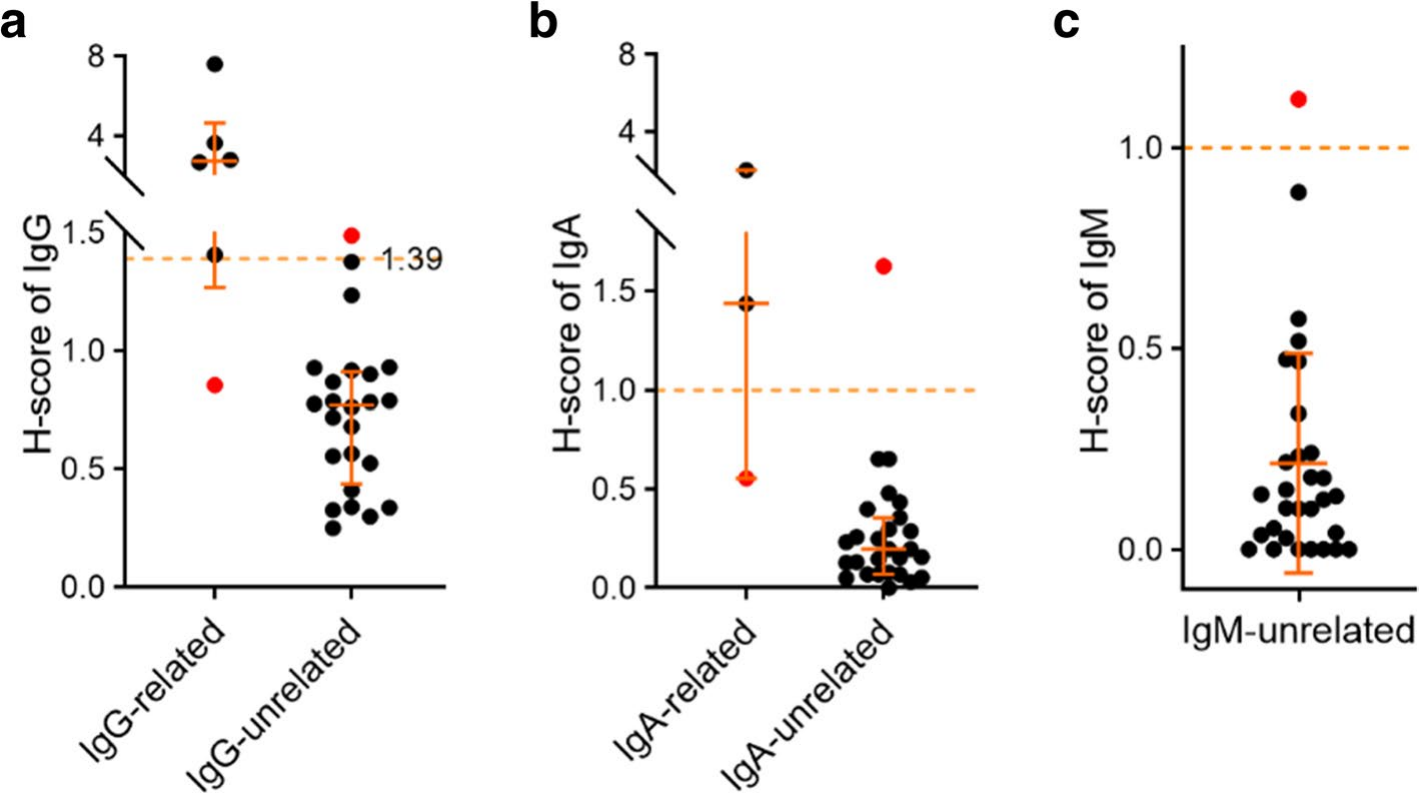
Analysis of the involvement of immunoglobulin heavy chain. **a, b.** Comparison of H-scores of IgG (**a**) and IgA (**b**) between their related and unrelated types. **c.** Distribution of IgM’s H-score in IgM unrelated cases. Median and interquartile range of the data is plotted

It is a great challenge to discriminate the participation of immunoglobulin heavy chains in amyloid deposition from AL case, especially for the involvement of IgG for its relatively high abundance in background. To conquer this problem, we introduced a second superiority score for immunoglobulin heavy chains (H-score) as illustrated in the material and methods section. The median value of IgG’s H-score in IgG unrelated cases was 0.77 (IQR, 0.50—0.90), which was much higher than that of IgA in IgA unrelated cases (0.20 (IQR, 0.08—0.33)) and IgM in IgM unrelated cases (0.13 (IQR, 0.03—0.24)) (Fig. 3a-c), showing its higher background signal. The cut-off value of IgG’s H-score for judging IgG’s involvement could be set to 1.39 that has the highest sensitivity and specificity (Fig. 3a), while that of IgA and IgM could be set to 1 for easy to analyze (Fig. 3b, c), though we did not identify IgM related cases to determine a sufficiently optimized cut-off value for it.

### Stepwise data interpretation procedure for amyloidosis typing

**Fig. 4:**
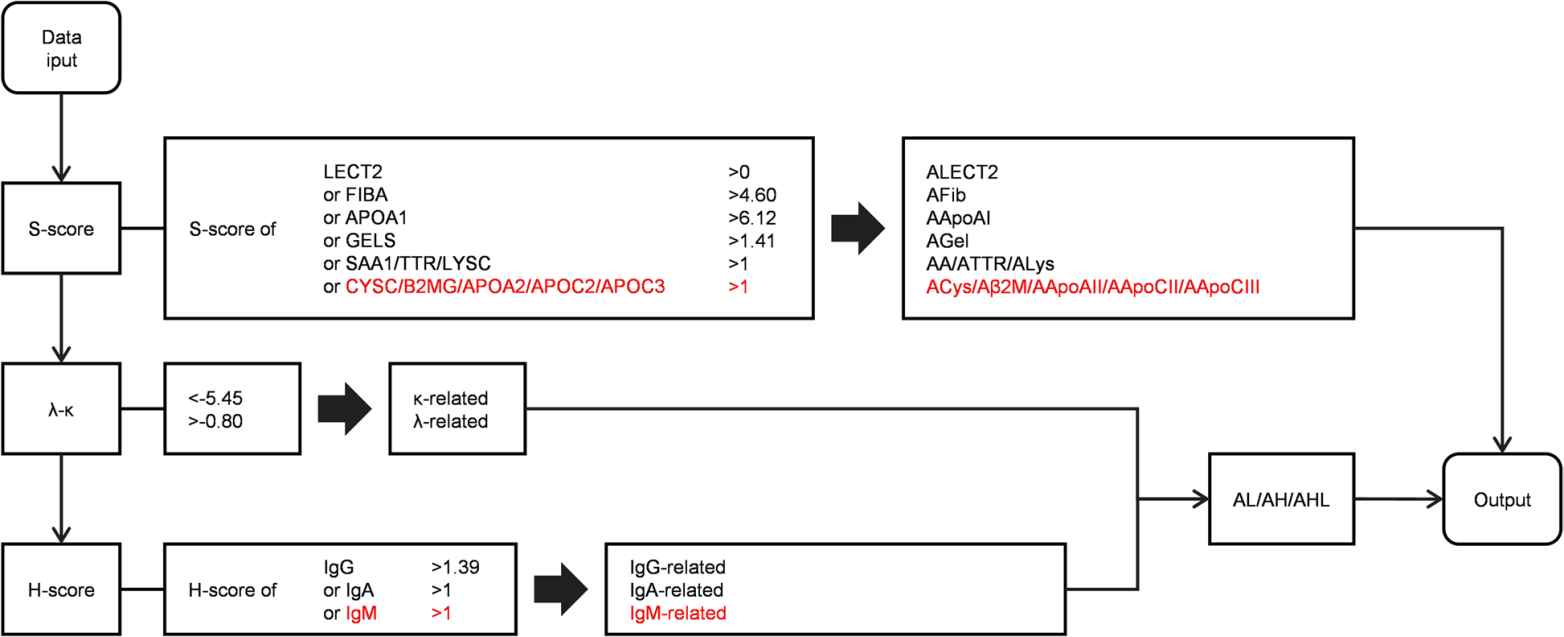
The stepwise data interpretation procedure for amyloidosis typing

Based on the characteristics of AFPs’ SC distribution in training dataset, we established a data interpretation procedure for amyloidosis typing as illustrated in Fig. 4. Firstly, the S-scores of AFPs other than immunoglobulin were analyzed, if any of the S-scores exclusively exceeds its cut-off value, the corresponding type would be determined. Secondly, for immunoglobulin amyloidosis, λ-κ value is analyzed to judge the involvement of immunoglobulin light chains. If λ-κ < -5.45, it is assigned to κ-related group; if λ-κ > -0.80, it is assigned to λ-related group. Thirdly, to judge the involvement of immunoglobulin heavy chains, H-scores of IgG/IgA/IgM were analyzed. If any of the H-scores exclusively exceeds its cut-off value, the corresponding immunoglobulin heavy chain’s involvement would be determined. If both immunoglobulin light and heavy chains’ involvement are recognized, it would be assigned to the corresponding AHLs accordingly.

### Performance of the amyloidosis typing procedure on validation dataset

The established procedure was then used to typing the 50 validation specimens, which achieved an 88% accuracy (Table 3). In detail, 3 ALECT2, 3 AGel, 2 AA, 1 ATTR, 12 ALκ, 15 ALλ, 2 AH (IgG), 4 AHL (IgGλ), 2 AHL (IgAλ) were successfully classified, and an ALκ and an ALλ failed to be identified, an ALλ and an AHL (IgGλ) were misclassified as AHL (IgGλ) and ALλ respectively, an AHL (IgAλ) was interfered by AHL (IgGλ) for both the IgA and IgG’s S-scores exceeded their thresholds, and an AH (IgG) was interfered by AH (IgM) for both IgG and IgM had high H-scores (Supplementary Table 1–4).

**Table 3:** LMD-MS typing results of validation set by utilizing the data interpretation process

Type							AH	AHL	
	ALECT2	AGel	AA	ATTR	ALκ	ALλ	IgG	IgGλ	IgAλ
Number	3	3	2	1	13	17	3	5	3
Correctly subtyped	3	3	2	1	12	15	2	4	2
Accuracy	100%	100%	100%	100%	92%	88%	67%	80%	67%
Total accuracy	88%								

### Diagnostic value for amyloidosis of accompanying proteins

**Fig. 5:**
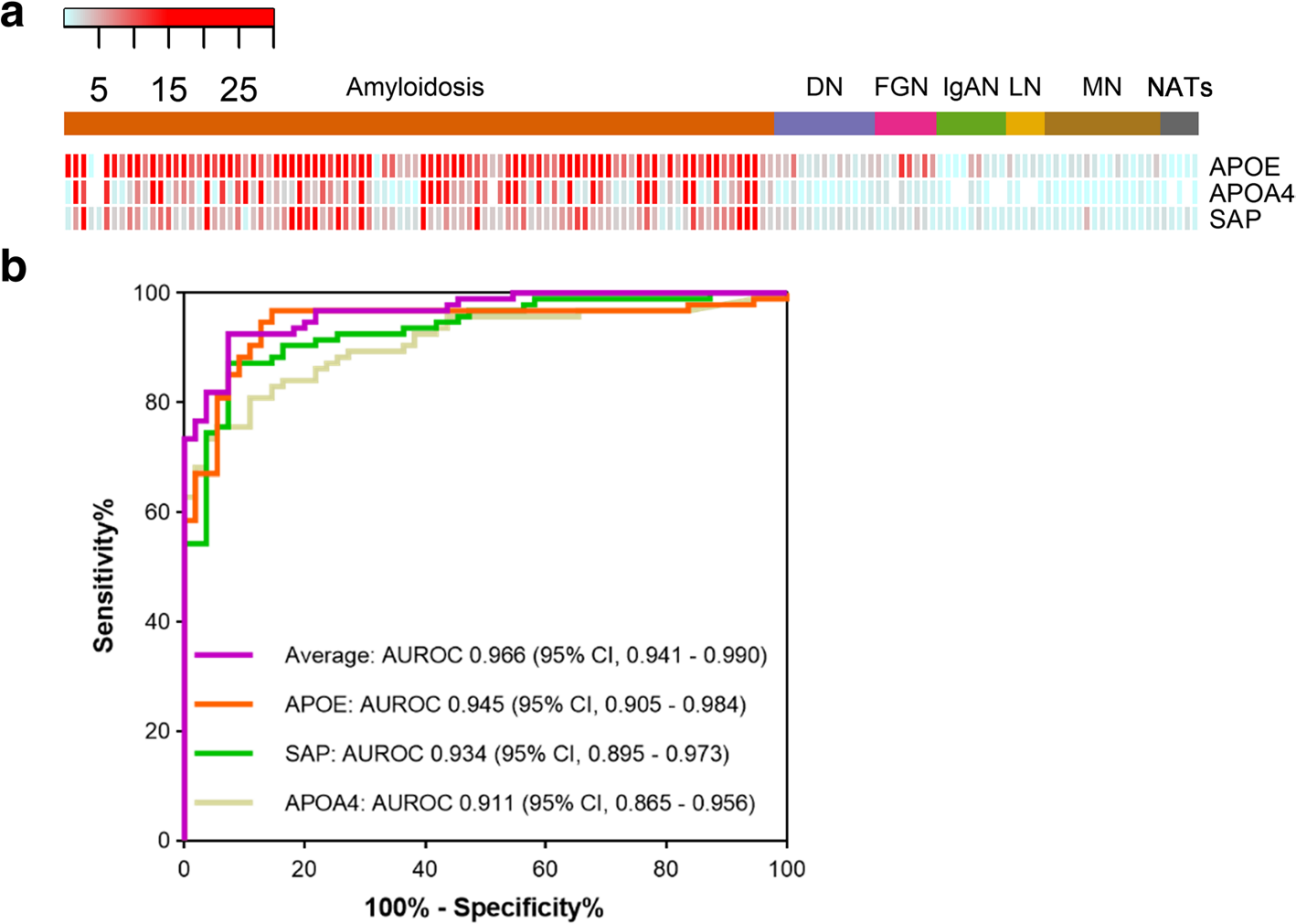
Diagnosis of amyloidosis by quantitative analysis of AAPs. **a.** Normalized SCs of the three AAPs in each amyloidosis and non-amyloid nephropathy sample are presented as heatmap. The type of each case is labeled by column, and protein name is labeled by row. **b.** Area under the receiver operating curve (AUROC) with respect to normalized SC of AAPs in amyloidosis versus non-amyloid nephropathy. All the three AAPs’ SC and their averaged value are used to generate the AUROC curve

As proposed by Vrana et al., the clinical diagnosis of amyloidosis by MS-based proteomics could be achieved by identifying at least two out of the three AAPs (APOE, APOA4 and SAP) [14]. In our experiment, we also observed that the AAPs were widely present at amyloid deposition sites (Fig. 5a). However, they were also always identified in glomerulus with other renal diseases, which indicate Vrana’s method would result in extremely high false positives. Nevertheless, these AAPs had a significant abundance superiority in amyloidosis specimens than in non-amyloid nephropathies. To establish a better diagnostic indicator, receiver operating curves (ROC) for normalized SC of each of the three AAPs and their average value were analyzed using the 92 amyloidosis specimens compared with 55 non-amyloid nephropathies. As expected, the average value showed the highest area under the ROC (AUROC) value of 0.966 (95% confidence interval [CI], 0.941–0.990). Among the single indicators, APOE had the best accuracy, with AUROC of 0.945 (95% CI, 0.905–0.984), followed by 0.934 (95% CI, 0.895–0.973) for SAP and 0.911 (95% CI, 0.865–0.956) for APOA4 (Fig. 5b).

## Discussion

Currently, the LMD-MS based amyloidosis typing is increasingly accepted as a clinical test. However, how to reading the MS data properly to make the right diagnosis is still a challenging for clinicians. In this study, we adopted a stepwise data interpretation procedure that includes or excludes the types group by group. 1) Other types besides immunoglobulin related amyloidosis were firstly confirmed or excluded by analyzing the S-scores; 2) followed by considering whether and which immunoglobulin light chain might be involved by analyzing λ-κ; and 3) whether and which immunoglobulin heavy chain participated in amyloid deposition was judged by analyzing the H-scores at last. This procedure was verified to have a total accuracy rate of 88% in validation phase. ALκ and ALλ are the main types, achieving accuracy of 92% and 88% respectively. The 100% accuracy in the less common types of ALECT2, AGel, AA and ATTR was achieved. But for immunoglobulin heavy chain related types, it varied from 67%-80%, that could be the most difficult systemic amyloidosis to type.

Of the 7 ALECT2 cases in this study, the SC of LECT2 was relatively low (absolute SC ranged from 3–16 and normalized SC ranged from 1.01–4.22), and were always lower than Igκ and IgG. This was similar to the results reported by Li et al. [22] and Mereuta et al. [29]. These results implied that the abundance of LECT2 may be underestimated by shotgun proteomics quantification. In addition, LECT2 was not identified in all other amyloidosis other than ALECT2 type and non-amyloid nephropathies. Therefore, ALECT2 can be determined by the identification of LECT2.

The major type of amyloidosis is AL, which is further subdivided into ALκ and ALλ. The background of Igκ was relatively high, especially in some ALλ cases, it was even slightly higher than Igλ. Here, we innovatively proposed the variable of λ-κ to distinguish the interference between Igκ and Igλ, and found that non-κ/λ related categories can be effectively identified by this variable (except for AFib and AApoAI, which could be recognized before λ-κ analysis). In validation dataset, there was only one AL (Igκ) case that had the λ-κ value (-4.06) a little higher than cutt-off (-5.45) and was not recognized immediately. Yet, none of the other types were assigned to this case. Other than that, the λ-κ analysis is precise for distinguishing κ, λ and non-κ/λ related cases.

Amyloid deposition of immunoglobulin heavy chain is usually accompanied with light chain. This brings a major challenge to distinguish AH and AHL from each other. Moreover, in some AL cases, the SC of IgG may have a higher value over light chains, which causes the confusion of AH, AHL and AL. Therefore, we introduced another score (H-score) to recognize the involvement of immunoglobulin heavy chain, especially for IgG. Of the total 51 AL specimens, 5 (9.8%) had higher IgG SC than light chains, which may be easily misclassified to IgG related types. This phenomenon was not observed for IgA and IgM. This may be due to the high background of IgG in the blood that causes interference, and thus the analysis for IgG should be different from IgA & IgM. Consequently, we found that the H-score cut-off for IgG was higher than IgA & IgM. Despite the utilization of H-score, several mistakes were generated (Fig. 3 and Table 3). Among which, the SC of IgG was overrepresented in an AL (Igλ) case and was underrepresented in an AHL (IgGλ) case, as well as in an AHL (IgAλ) case, IgG and IgA both exceeded their cut-off, and in an AH (IgG) case, contradiction happened between IgG and IgM. These mistakes were all associated with IgG, requiring the typing method to be further improved in this respect. Also, these cut-off values may need to be optimized by more clinical specimens in future study. For now, it might be better to take a look at histopathological or hematological test results and rely on experienced clinicopathologists to determine whether and which heavy chain is involved. Nevertheless, these mistakes have little influence on the formulation of clinical treatment plan.

Qualitative analysis of AAPs is not an appropriate method to diagnose amyloidosis, since it could not exclude other renal diseases. This may be mainly because of the development of MS technology, which becomes more sensitive and can identify more proteins at lower abundance. Here, we proposed the quantitative analysis way that use the average abundance of the three AAPs, and indicated it as a fine indicator to diagnose amyloidosis.

For AL amyloidosis, it is desirable to assess the clonal disorder in serum and urine, and for hereditary amyloidosis, genotyping analysis is pertinent to confirm the typing result. Nevertheless, we failed to collect enough data in these respects, which are limitations of the present study, however, relevant analyses should be addressed when possible. There were still several rare types such as ACys, Aβ2M, AApoAII, AApoCII and AApoCIII that were not included in this study. This was mainly because of the low incidence of systemic amyloidosis (10 per million) and even lower for rare types. The data interpretation process will be further verified and optimized in future clinical tests and follow-up studies, as well as the MS data characteristics of these not included types are to be determined more detailly in the future.

## Conclusions

This study demonstrates the value of LMD-MS technique for precise diagnosis of amyloidosis. The principle and details of the stepwise process of MS data analysis that can improve typing accuracy was proposed for the first time, where some cut-off values are defined and set. This scheme includes not only the diagnosis of AL, but also relatively rare subtypes, which has a high clinical practical value. The development of this method will be helpful for clinicians to accurately typing amyloidosis. In addition, we discovered that the quantitative analysis of the AAPs could be a fine indicator to identify amyloidosis from other nephropathies, which is valuable to be confirmed and developed as diagnostic indicator.

## Supplementary Information

**Additional file 1: Supplementary Figure 1.** Analysis of SC of amyloid proteins with high background abundance.

**Additional file 2: Supplementary Figure 2.** Analysis of S-score of typical amyloid proteins with relatively clean background.

**Additional file 3: Supplementary Table 1.** Normalized SC of amyloid proteins of validation specimens.

**Additional file 4: Supplementary Table 2.** S-score calculation in validation set.

**Additional file 5: Supplementary Table 3.** λ-κ calculation of immunoglobulin related amyloidosis in validation set.

**Additional file 6: Supplementary Table 4.** H-score calculation of immunoglobulin related amyloidosis in validation set.

## Data Availability

Data supporting the results are reported in this article and additional information is available. In addition, relevant materials used in the study are available from the corresponding author on reasonable request.

## Abbreviations

AA: Serum amyloid A amyloidosis
AApoAII: Apolipoprotein A-II Amyloidosis
AApoCII: Apolipoprotein C-II Amyloidosis
AApoCIII: Apolipoprotein C-III Amyloidosis
Aβ2M: Beta-2-microglobulin Amyloidosis
ACys: Cystatin-C amyloidosis
AFib: Fibrinogen α chain amyloidosis
AGel: Gelsolin amyloidosis
AH: Immunoglobulin heavy chain amyloidosis
AHL: Immunoglobulin heavy-light chain amyloidosis
AL: Immunoglobulin light chain amyloidosis
ALECT2: Leukocyte chemotactic factor-2 amyloidosis
ALκ: AL, κ-type
ALλ: AL, λ-type
APOA1: Apolipoprotein A-I
APOA2: Apolipoprotein A-II
APOA4: Apolipoprotein A-IV
APOC2: Apolipoprotein C-II
APOC3: Apolipoprotein C-III
APOE: Apolipoprotein E
ATTR: Transthyretin amyloidosis
AUROC: Area under receiver operating curve
CR: Congo red
B2MG: Beta-2-microglobulin
CYSC: Cystatin-C
DN: Diabetic nephropathy
FFPE: Formalin fixed paraffin-embedded
FGN: Fibrillary glomerulonephritis
FIBA: Fibrinogen A α chain
FIBB: Fibrinogen B β chain
FIBG: Fibrinogen G γ chain
FLC: Free light chain
H-score: Superiority of immunoglobulin heavy chain over light chain
IFE: Immunofixation electrophoresis
IgAN: IgA nephropathy
IQR: Interquartile range
LMD-MS: Laser microdissection combined with mass spectrometry
LN: Lupus nephritis
LYSC: Lysozyme C
MN: Membranous nephropathy
NATs: Normal tissue adjacent to tumors
SAA1: Serum amyloid A-1
SAP: Serum amyloid P component
SC: Spectral count
S-score: Superiority score of AFPs other than immunoglobulin
TTR: Transthyretin
