# Supplementary figures and images for: A stepwise data interpretation process for renal amyloidosis typing by LMD-MS
Source: BMC Nephrol. 2022 Apr 13;23:144. doi: 10.1186/s12882-022-02785-9 (PMC9008935; doi:10.1186/s12882-022-02785-9)

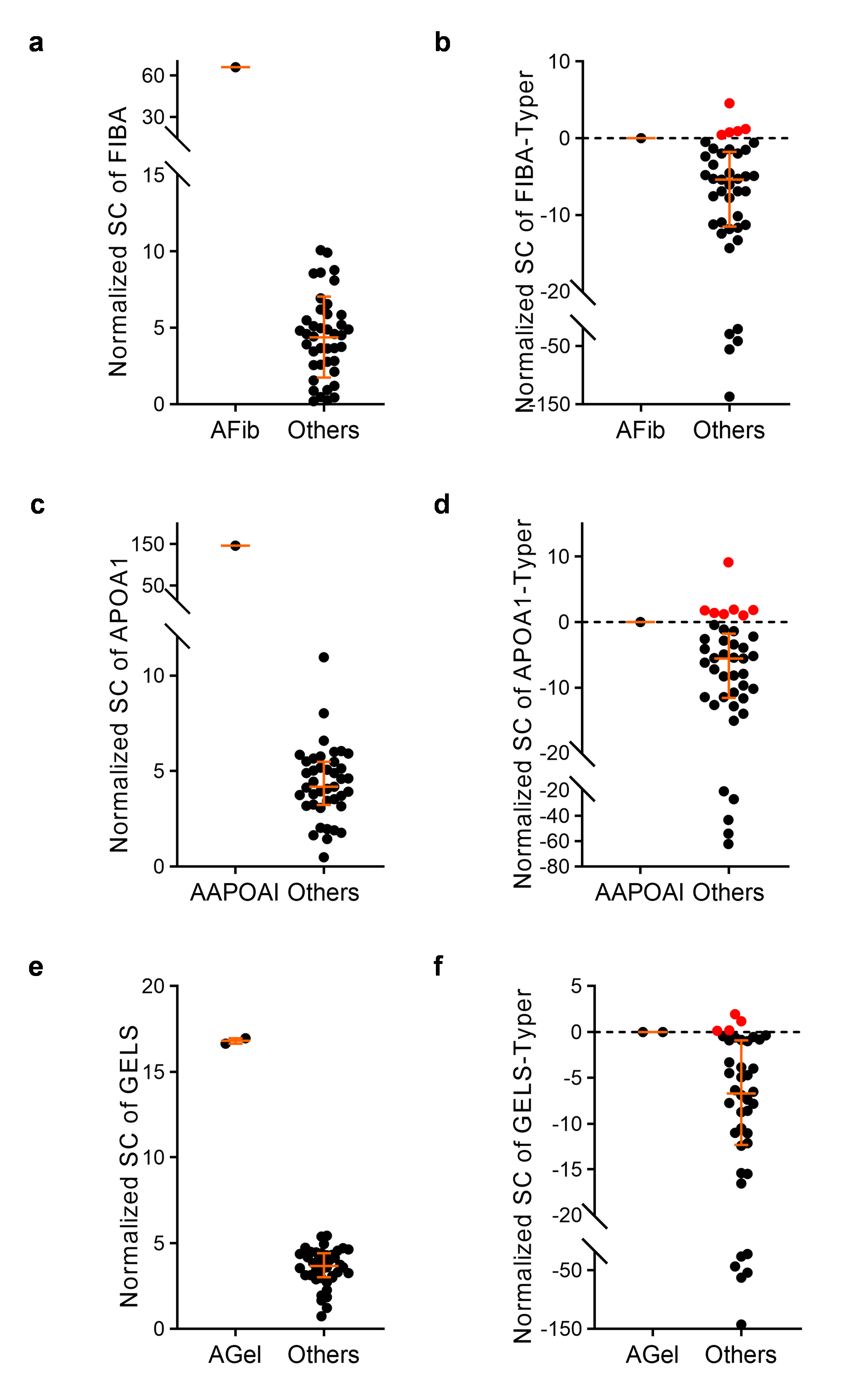

Supplement: Supplementary file 1 — Additional file 1: Supplementary Figure 1. Analysis of SC of amyloid proteins with high background abundance. [file 12882_2022_2785_MOESM1_ESM.tif]

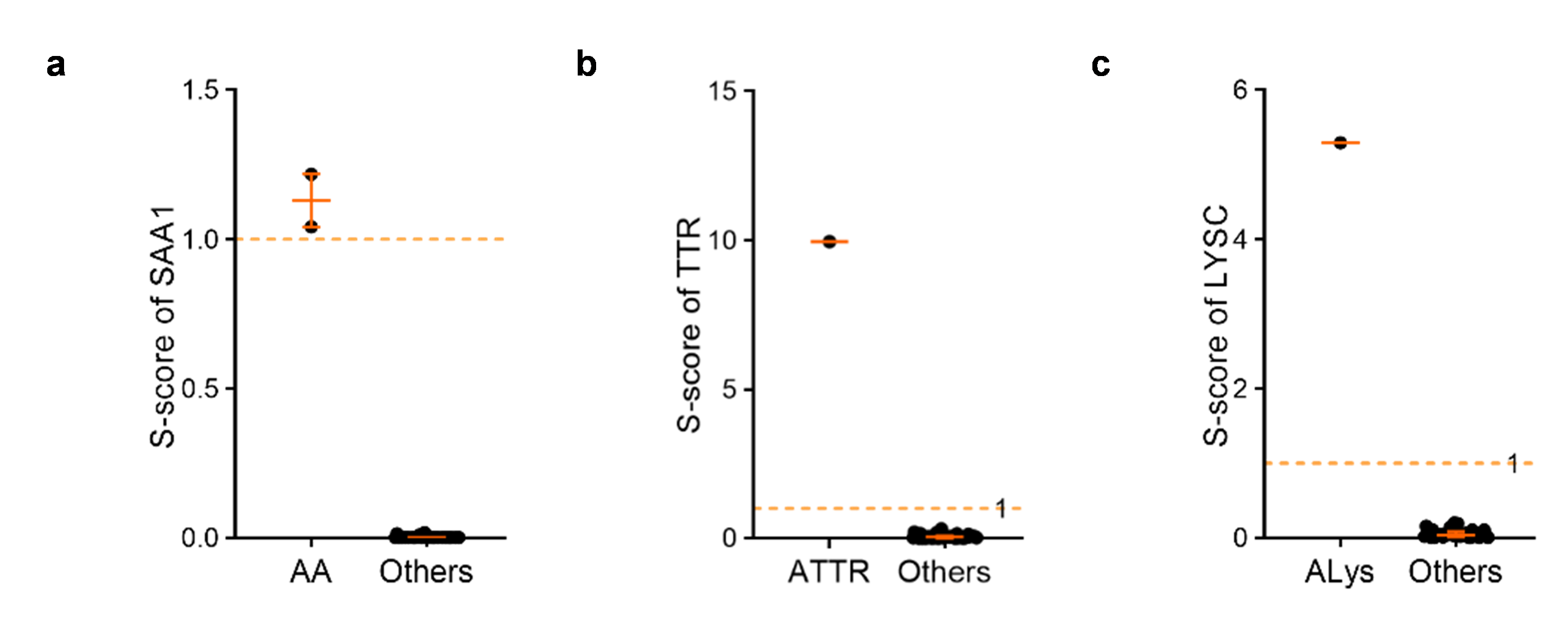

Supplement: Supplementary file 2 — Additional file 2: Supplementary Figure 2. Analysis of S-score of typical amyloid proteins with relatively clean background. [file 12882_2022_2785_MOESM2_ESM.tif]
